# Spectroscopic Properties of Er^3+^-Doped Particles-Containing Phosphate Glasses Fabricated Using the Direct Doping Method

**DOI:** 10.3390/ma12010129

**Published:** 2019-01-03

**Authors:** Pablo Lopez-Iscoa, Nirajan Ojha, Ujjwal Aryal, Diego Pugliese, Nadia G. Boetti, Daniel Milanese, Laeticia Petit

**Affiliations:** 1 Dipartimento di Scienza Applicata e Tecnologia (DISAT) and INSTM UdR Torino Politecnico, Politecnico di Torino, Corso Duca degli Abruzzi 24, 10129 Torino, Italy; pablo.lopeziscoa@polito.it (P.L.-I.); diego.pugliese@polito.it (D.P.); daniel.milanese@polito.it (D.M.); 2 Laboratory of Photonics, Tampere University, Korkeakoulunkatu 3, 33720 Tampere, Finland; nirajan.ojha@tut.fi (N.O.); ujjwal.aryal@student.tut.fi (U.A.); 3 Fondazione LINKS—Leading Innovation & Knowledge for Society, Via P. C. Boggio 61, 10138 Torino, Italy; boetti@ismb.it; 4 Istituto di Fotonica e Nanotecnologie-Consiglio Nazionale delle Ricerche (IFN-CNR), Caratterizzazione e Sviluppo di Materiali per la Fotonica e l’Optoelettronica (CSMFO) Lab., Via alla Cascata 56/C, 38123 Povo (TN), Italy; 5 nLIGHT Corporation, Sorronrinne 9, 08500 Lohja, Finland

**Keywords:** phosphate glass, oxyfluoride phosphate glass, Er_2_O_3_-doped particles, direct particle doping, Er^3+^ luminescence property

## Abstract

The effect of the incorporation of Er_2_O_3_-doped particles on the structural and luminescence properties of phosphate glasses was investigated. A series of different Er_2_O_3_-doped TiO_2_, ZnO, and ZrO_2_ microparticles was synthesized using soft chemistry and then added into various phosphate glasses after the melting at a lower temperature than the melting temperature. The compositional, morphological, and structural analyses of the particles-containing glasses were performed using elemental mapping by field emission-scanning electron microscopy (FE-SEM) with energy dispersive x-ray spectrometry (EDS) and x-ray diffraction (XRD). Additionally, the luminescence spectra and the lifetime values were measured to study the influence of the particles incorporation on the spectroscopic properties of the glasses. From the spectroscopic properties of the glasses with the composition 50P_2_O_5_-40SrO-10Na_2_O, a large amount of the Er_2_O_3_-doped particles is thought to dissolve during the glass melting. Conversely, the particles were found to survive in glasses with a composition 90NaPO_3_-(10 − x)Na_2_O-xNaF (with x = 0 and 10 mol %) due to their lower processing temperature, thus clearly showing that the direct doping method is a promising technique for the development of new active glasses.

## 1. Introduction

Among rare-earth (RE) ions, erbium (Er^3+^) ions have been widely studied as dopants in different host matrices due to their emission at around 1.5 µm, which makes them suitable for applications such as fiber lasers and amplifiers for telecommunications [1]. Moreover, their up-conversion properties enable them to convert the infrared (IR) radiation into red and green emissions. Thus, Er^3+^-doped materials show many other applications such as photovoltaics [2], display technologies [3], and medical diagnostics [4].

Phosphate glasses are very well known for their suitable mechanical and chemical properties, homogeneity, good thermal stability, and excellent optical properties [5,6,7,8,9]. As compared to silicate glasses, they possess low glass transition (*T_g_*) (400–700 °C) and crystallization (*T_p_*) (800–1400 °C) temperatures, which facilitate their processing and fabrication by melt-quenching technique [10,11]. Phosphate glasses also exhibit high RE ions solubility [12], leading to quenching phenomenon occurring at very high concentrations of RE [9,13]. Due to their outstanding optical properties, RE-doped phosphate glasses have recently become appealing for the engineering of photonic devices for optical communications [14], laser sources, and optical amplifiers [9,13,15,16,17]. Moreover, due to their low phonon energies and wide optical transmission window from Ultraviolet (UV) to mid-IR regions, fluoride phosphate glasses are considered to be good glass candidates for Er^3+^ doping [18].

It is well known that the luminescence properties as well as the solubility of RE ions in glassy hosts can be significantly impacted by parameters such as the mass, covalency or charge of the ligand atoms [19]. The luminescence properties of Er^3+^-doped phosphate glasses with compositions in mol % of (0.25 Er_2_O_3_2013(0.5 P_2_O_5_ − 0.4 SrO − 0.1 Na_2_O)_10−x_ − (TiO_2_/Al_2_O_3_/ZnO)_x_), with x = 0 and 1.5 mol %, were previously investigated and discussed as a function of the glass composition [20]. It was found that the Er^3+^ ions spectroscopic properties depended on the glass structure connectivity, which changed the Er^3+^ ions solubility. The intensity of the emission at 1.5 µm could be increased if the phosphate network is depolymerized. Recently, the impact of the nucleation and growth on the Er^3+^ spectroscopic properties of these glasses was reported in Reference [21]. Indeed, due to the crystalline environment surrounding the RE ions, the RE-doped glass-ceramics have shown to combine glass properties (large flexibility of composition and geometry) with some advantages of the RE-doped single crystals (higher absorption, emission, and lifetimes) [22]. Upon heat treatment, the crystals precipitated from the surface of the glasses and the composition of the crystals depended on the glass composition. The crystals were found to be Er^3+^ free except in the glass with x = 0. With this study, it was shown that the heat treatment does not necessarily lead to the bulk precipitation of Er^3+^-doped crystals, which should increase the spectroscopic properties of the glass.

Therefore, a new route was developed in order to control the local environment of the RE ions independently of the glass composition: the direct doping method [23]. With this technique, the particle matrix allows high RE content in a high dispersion state, thus avoiding the quenching effect independently of the glass composition [24]. Thus, innovative particles-containing glasses with specific particle compositions and nanostructures can be achieved by adding RE-doped particles in the glass batch after the melting process. Recently, up-conversion (UC) was obtained from phosphate glasses which contain only 0.01 at % of Er^3+^ and 0.06 at % of Yb^3+^ by adding NaYF_4_:Er^3+^, Yb^3+^ nanoparticles (NPs) in the glass after the melting [25]. However, as explained in Reference [26], the main challenge with this novel route of preparing glasses is to balance the survival and dispersion of the particles in the glasses. The particles should be thermally stable at the temperature they are added in the glass melt to ensure their survival within the glass during the glass preparation.

Here, new Er^3+^-doped particles-containing phosphate glasses were prepared in order to fabricate phosphate glasses with enhanced emission at 1.5 µm as compared to standard Er^3+^-doped phosphate glasses. Oxyfluoride phosphate glass family was also considered due to its low processing temperature. At first, the synthesis and characterization of different Er_2_O_3_-doped particles are presented. The concentration of Er_2_O_3_ in the particles was kept high to ensure that luminescence can be detected after embedding the particles into the glasses. The particles were prepared by the sol-gel method. TiO_2_, ZnO, and ZrO_2_ were selected as crystalline hosts for the Er^3+^ ions due to their high melting points and low phonon energies, which provide high thermal stability and high luminescence properties [27,28,29,30,31,32]. Then, the technique of incorporation of the particles into phosphate glasses with various compositions was addressed. Finally, a full characterization of the morphological and luminescence properties of the different particles-containing glasses was reported.

## 2. Materials and Methods

### 2.1. Particles Synthesis

The synthesis of the TiO_2_ particles doped with 14.3 mol % of Er_2_O_3_ was reported in Reference [33]. Specifically, 21.28 g of titanium(IV) butoxide reagent grade (97% Sigma–Aldrich, Saint Louis, MO, USA) were dissolved in ethanol (100 mL) and then added dropwise into a mixture of deionized water (2 mL), ethanol (>99.8% Sigma–Aldrich, Saint Louis, MO, USA), and 8.37 g of erbium (III) acetate (>99.9% Sigma–Aldrich, Saint Louis, MO, USA). Once the addition was completed, the solution was heated at a reflux temperature of 90 °C and left under reflux for 1 day. Finally, the precipitates were collected by centrifugation, washed with ethanol for several times, and dried at 100 °C for 1 day. The as-prepared sample was further annealed in air at 800 °C for 2 h.

The ZnO particles were synthesized following the process described in Reference [34]. The concentration of Er_2_O_3_ was 14.3 mol % as in the TiO_2_ particles. Then, 13.5 g of zinc acetate dihydrate (Zn(CH_3_COO)_2_·2H_2_O)) (99.999% Sigma–Aldrich, Saint Louis, MO, USA) and 7.8 g of erbium chloride hexahydrate (ErCl_3_ 6H_2_O) (Sigma–Aldrich, Saint Louis, MO, USA, 99.9% purity) were used as starting materials. The precursors were dissolved in ethanol (>99.8% Sigma–Aldrich, Saint Louis, MO, USA)-deionized water (50–50% in volume) with polyvinylpyrrolidone (PVP K 30, average Mw 40,000, Sigma–Aldrich, Saint Louis, MO, USA) as a surfactant and stirred for 30 min until a clear solution was formed. Then, the sodium hydroxide 0.1 M (Fluka Massachusetts, MA, USA) was added until reaching a pH of 9, which is optimal for nucleation. The solution was heated up to 90 °C and stirred for 4 h to get fine precipitation. The obtained precipitation was washed 4 times with deionized water and centrifuged. The precipitation was collected and dried at 80 °C for 4 h. Finally, the sample was calcined at 1000 °C for 2 h.

The ZrO_2_ particles were synthesized with 7 mol % of Er_2_O_3_ as in Reference [35]. More in detail, 28 mL of aqueous solution of 0.1 M erbium chloride hexahydrate (ErCl_3_·6H_2_O, 99.9%, Sigma Aldrich, Saint Louis, MO, USA), and 1.83 mL of 0.1 M sodium bicarbonate (NaHCO_3_, 99.7%, Fluka) were added to 50 mL of absolute ethanol (>99.8% Sigma Aldrich, Saint Louis, MO, USA) while stirring at 60 °C. Then, 9.17 mL of zirconium (IV) butoxide solution ((Zr(OBu)_4_) 80 wt % in 1-butanol) (Sigma-Aldrich, Saint Louis, MO, USA) was added and kept under stirring for 2 h. The obtained colloidal solution was centrifuged and washed three times with ethanol (>99.8% Sigma–Aldrich, Saint Louis, MO, USA) at 9000 rpm for 30 min. Lastly, the final product was calcined at 1000 °C for 2 h.

### 2.2. Particles-Containing Glasses Preparation

The particles-containing glasses were prepared by incorporating the aforementioned particles in the host glasses with compositions (in mol %): 50P_2_O_5_-40SrO-10Na_2_O, labeled as SrNaP glass, and 90NaPO_3_-(10 − x)Na_2_O-xNaF with x = 0 and 10, labeled as NaPF0 and NaPF10, respectively. The glasses were synthesized in a quartz crucible using Na_6_O_18_P_6_ (Alfa-Aesar, technical grade), Na_2_CO_3_ (Sigma–Aldrich, >99.5%), Sr(PO_3_)_2_, and NaF (Sigma–Aldrich, 99.99%). Sr(PO_3_)_2_ precursor was independently prepared using SrCO_3_ (Sigma-Aldrich, Saint Louis, MO, USA, ≥99.9%), and (NH_4_)_2_HPO_4_ as raw materials heated up to 850 °C. Prior to the melting, the glass NaPF0 was heat treated at 400 °C for 30 min to decompose Na_2_CO_3_ and evaporate CO_2_. Details on the direct doping process of the NaPF0 and NaPF10 glasses and of the SrNaP glass can be found in References [26] and [36], respectively. The particles (1.25 wt %) were incorporated in the SrNaP glass at 1000 °C after melting at 1050 °C for 20 min. After 5 min, the glasses were quenched. The NaPF0 and NaPF10 glasses were melted at 750 °C for 5 min, then the particles were added at 550 °C and finally the glass melts were quenched after a 3 min dwell time.

After quenching, all the melts were finally annealed at 40 °C below their respective *T_g_* for 5 h in order to release the residual stress. All the glasses were cut and optically polished or ground, depending on the characterization technique.

### 2.3. Characterization Techniques

The thermal stability of the particles was measured by thermogravimetric analysis (TGA) using a Perkin Elmer TGS-2 instrument (PerkinElmer Inc., Waltham, MA, USA). The measurement was carried out in a Pt crucible at a heating rate of 10 °C/min in a controlled atmosphere (N_2_ flow), featuring an error of ± 3 °C. The sample was approximately 10 mg of grounded particles.

The composition and morphology of the particles were determined using a field emission scanning electron microscope (FE-SEM, Zeiss Merlin 4248, Carl Zeiss, Oberkochen, Germany) equipped with an Oxford Instruments X-ACT detector and energy dispersive spectroscopy systems (EDS) (Oxford Instruments, Abingdon-on-Thames, UK). The composition and morphology of the particles-containing glasses were determined using FE-SEM/EDS (Carl Zeiss Crossbeam 540 equipped with Oxford Instruments X-Max^N^ 80 EDS detector) (Instruments, Abingdon-on-Thames, UK). The images were taken at the surface of the glasses, previously cut and optically polished. The samples were coated with a thin carbon layer before the EDS elemental mapping. The elemental mapping analysis of the composition of the samples was performed by using the EDS within the accuracy of the measurement (± 1.5 mol %).

The crystalline phases were identified using the X-ray diffraction (XRD) analyzer Philips X’pert (Philips, Amsterdam, Netherlands) with Cu K_α_ X-ray radiation (λ = 1.5418 Å). Data were collected from 2θ = 0 up to 60° with a step size of 0.003°.

The emission spectra in the 1400–1700 nm range were measured with a Jobin Yvon iHR320 spectrometer (Horiba Jobin Yvon SAS, Unterhaching, Germany) equipped with a Hamamatsu P4631-02 detector (Hamamatsu Photonics K.K., Hamamatsu, Japan) and a filter (Thorlabs FEL 1500, Thorlabs Inc., Newton, NJ, USA). Emission spectra were obtained at room temperature using an excitation monochromatic source at 976 nm, emitted by a single-mode fiber pigtailed laser diode (CM962UF76P-10R, Oclaro Inc., San Jose, CA, USA).

The fluorescence lifetime of Er^3+^:^4^I_13/2_ energy level was obtained by exciting the samples with a fiber pigtailed laser diode operating at the wavelength of 976 nm, recording the signal using a digital oscilloscope (Tektronix TDS350, Tektronic Inc., Beaverton, OR, USA) and fitting the decay traces by single exponential. All lifetime measurements were collected by exciting the samples at their very edge to minimize reabsorption. Estimated error of the measurement was ±0.2 ms. The detector used for this measurement was a Thorlabs PDA10CS-EC (Thorlabs Inc., Newton, NJ, USA). The samples used for the spectroscopic measurements were previously cut and optically polished.

## 3. Results and Discussion

**Figure 1:**
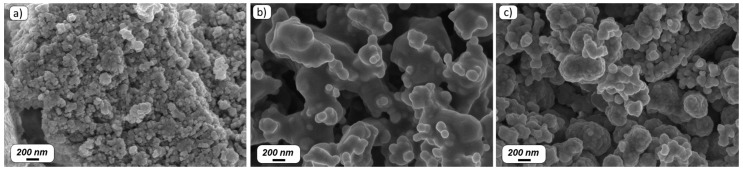
FE-SEM micrographs of Er_2_O_3_-doped TiO_2_ (**a**), ZnO (**b**), and ZrO_2_ (**c**) particles.

Er_2_O_3_-doped TiO_2_, ZnO, and ZrO_2_ particles were synthesized using the sol-gel technique. The morphology of the particles was assessed by FE-SEM analysis (see Figure 1).

The Er^3+^-doped particles show agglomerates of nanoparticles with irregular shape. As can be seen from Figure 1, the agglomerates are formed by small particles with a size of ~50–100 nm for the TiO_2_, ~100–400 nm for the ZnO, and ~100–300 nm for the ZrO_2_ particles. Additionally, based on the EDS analysis, the composition of the particles was in agreement with the nominal one within the accuracy of the measurement.

**Figure 2:**
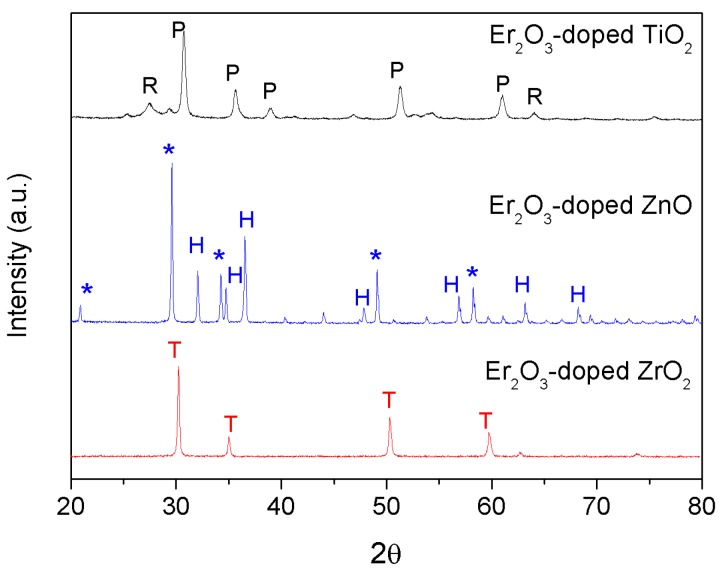
XRD patterns of the Er_2_O_3_-doped TiO_2_, ZnO, and ZrO_2_ particles, with the reference patterns of the pyrochlore (P), rutile (R), hexagonal (H), Zn extra phase (*), and tetragonal (T) crystalline phases.

The XRD patterns of the Er^3+^-doped particles are presented in Figure 2.

The XRD pattern of the Er_2_O_3_-doped TiO_2_ particles showed the presence of rutile phase (Inorganic Crystal Structure Database (ICSD) file No. 00-021-1276) and also the pyrochlore phase (ICSD file No. 01-073-1647), which seems to be the major phase as reported in Reference [37] when adding Er_2_O_3_. The XRD pattern of the Er_2_O_3_-doped ZnO particles revealed the presence of ZnO hexagonal wurtzite phase (ICSD file No. 89-1397), together with an extra unidentified phase. Similar results were reported by Rita John et al. [29], where the same unidentified phase was found in the XRD analysis of highly Er_2_O_3_-doped ZnO particles. The ZrO_2_ tetragonal crystalline phase (ICSD file No. 66787) is present in the Er_2_O_3_-doped ZrO_2_ sample, in agreement with References [38,39]. It should be pointed out that no peaks related to Er_2_O_3_ were observed in the XRD measurements of all the samples.

Additionally, the fluorescence lifetime values of the Er^3+^:^4^I_13/2_ level upon 976 nm excitation of the Er_2_O_3_-doped TiO_2_ and Zr_2_O particles were (0.1 ± 0.2) and (1.0 ± 0.2) ms, respectively. However, in the case of the ZnO particles, the lifetime value could not be measured due to low IR emission from the particles. The high concentration of Er_2_O_3_ in the particles is expected to lead to concentration quenching resulting in a very short lifetime [9].

**Figure 3:**
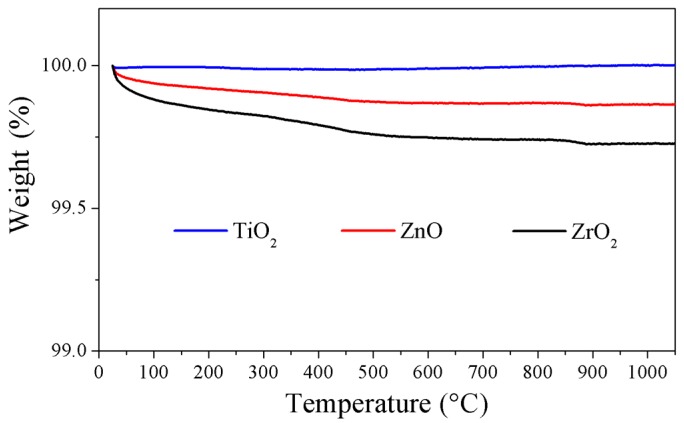
TGA analysis of the Er_2_O_3_-doped TiO_2_, ZnO, and ZrO_2_ particles.

The thermal stability of the particles was assessed by TGA analysis (see Figure 3).

Less than 1% of weight loss from all the investigated particles was detected when the temperature increased to 1050 °C, thus indicating that the particles should not suffer any degradation when added to the glass melt.

The SrNaP glasses were fabricated by incorporating the Er_2_O_3_-doped particles at 1000 °C after the melting as in References [36,40]. The ZnO and ZrO_2_ particles-containing SrNaP glasses exhibited a slight pink coloration, while TiO_2_ particles-containing glass displayed a purple color. Agglomerates of particles were found in the glasses by naked eyes. No diffraction peaks of any crystalline phase were found in the particles-containing glasses (data not shown), confirming that the number of particles in the glasses was too small to be detected.

**Figure 4:**
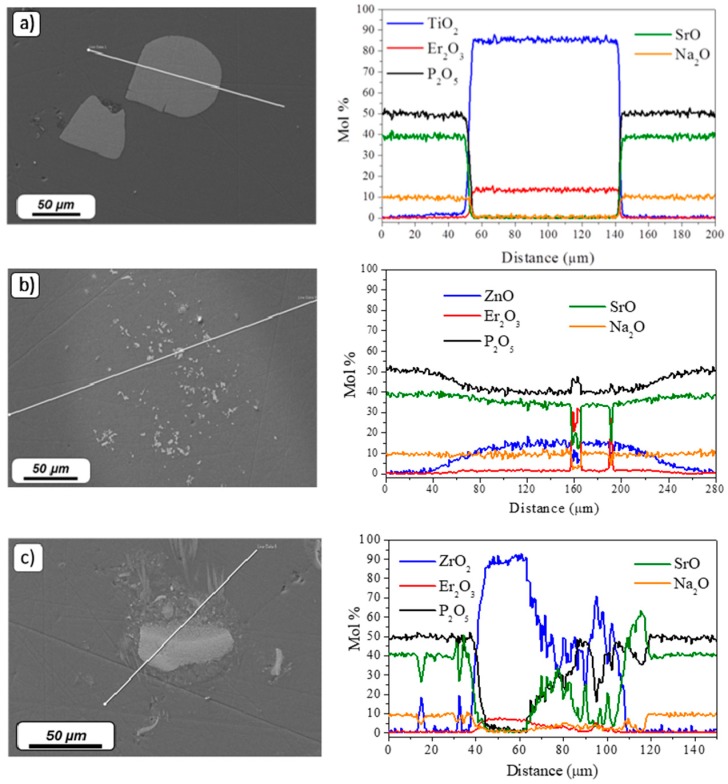
FE-SEM pictures with their corresponding elemental mapping of Er_2_O_3_-doped TiO_2_ (**a**), ZnO (**b**), and ZrO_2_ (**c**) particles-containing SrNaP glasses. The direction of the scan starts at the circle of the white line (corresponding to 0 µm).

The morphology and the composition of the particles found at the surface of the glasses were analyzed using FE-SEM/EDS analysis. The FE-SEM pictures and elemental mapping of Er_2_O_3_-doped TiO_2_, ZnO, and ZrO_2_ particles found in the SrNaP glasses are shown in Figure 4a–c, respectively.

Few microparticles with a size of ~100 µm were found in the Er_2_O_3_-doped TiO_2_ particles-containing glass matrix. The composition of the glass matrix and of the particles are in accordance with the theoretical ones. The remaining TiO_2_ particles maintained their compositional integrity in their center after the melting process, confirming the survival of some Er_2_O_3_-doped TiO_2_ particles in the glass. However, most of the Er_2_O_3_-doped TiO_2_ particles are suspected to degrade during the glass preparation. Regarding the Er_2_O_3_-doped ZnO particles-containing glasses, a bright area rich in Zn surrounds small particles with a size of ~15 µm. These particles contain mostly Er and P, thus indicating that Zn^3+^ ions diffused from the particles into the glass matrix during the glass preparation leading most probably to the precipitation of ErPO_4_ crystals (see Figure 4b). Therefore, the Er_2_O_3_-doped ZnO particles are also expected to degrade in the glass during its preparation, leading to the diffusion of Zn and Er in the glass matrix which is in agreement with the pink coloration of the glass itself. Figure 4c shows the mapping of the Er_2_O_3_-doped ZrO_2_ particles-containing glasses. Particles with a size of ~50 µm were found. These particles also maintained their compositional integrity in their center. However, contrary to the other particles, Sr-rich crystals were observed around the particles as seen in Reference [36], confirming that crystals with specific composition such as ZrO_2_ and SrAl_2_O_4_ precipitated due to the decomposition of the particles.

**Figure 5:**
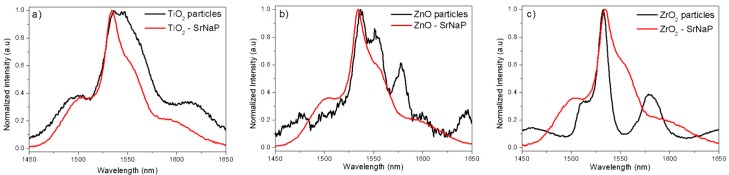
Emission spectra of the Er_2_O_3_-doped TiO_2_ (**a**), ZnO (**b**), and ZrO_2_ (**c**) particles and of their corresponding particles-containing SrNaP glasses.

The normalized emission spectra of the Er_2_O_3_-doped TiO_2_, ZnO, and ZrO_2_ particles and of their corresponding particles-containing SrNaP glasses are reported in Figure 5.

The particles-containing glasses exhibit a different emission shape compared to that of the particles alone, indicating that the neighboring ions arrangement around the Er^3+^ ions is different after embedding the particles in the glasses. The emission spectra of the glasses are typical of the emission band assigned to the Er^3+^transition from ^4^I_13/2_ to ^4^I_15/2_ in glass. Additionally, the investigated glasses show similar emission in shape, indicating that the environment of Er^3+^ ions is similar in the investigated glasses, as suspected from the Er^3+^:^4^I_13/2_ lifetime values measured at 976 nm (~4.0 ± 0.2 ms) in the three investigated glasses. It should be pointed out that these lifetimes are longer than the lifetime of the particles alone. Therefore, the absence of sharp peaks in the IR emission spectra together with the long Er^3+^:^4^I_13/2_ lifetime values and the absence of up-conversion emission from the glasses clearly suggest that a large number of particles suffered an important degradation during the melting process. This experimental evidence is in agreement with the color of the glasses, as reported in References [26,36,40]. Therefore, the Er^3+^ ions environment is expected to change from crystalline to glassy when the particles are incorporated into the glasses increasing the bandwidth of the emission band and the Er^3+^:^4^I_13/2_ lifetime.

In order to limit the degradation of the particles during the glass preparation, new glasses with a lower melting temperature and with a composition of 90NaPO_3_-(10 − x)Na_2_O-xNaF, with x = 0 (NaPF0) and 10 (NaPF10) (in mol %), were investigated as an alternative host for the Er_2_O_3_-doped particles. The optimization of the direct doping method can be found in Reference [25]; the Er_2_O_3_-doped particles were added at 550 °C and the dwell time was 3 min. As opposed to the NaSrP glasses, the Er_2_O_3_-doped TiO_2_- and ZnO-containing glasses exhibited a light pink coloration, whereas the Er_2_O_3_-doped ZrO_2_-containing glasses were colorless. Some agglomerates could be observed in the glasses with naked eye.

**Figure 6:**
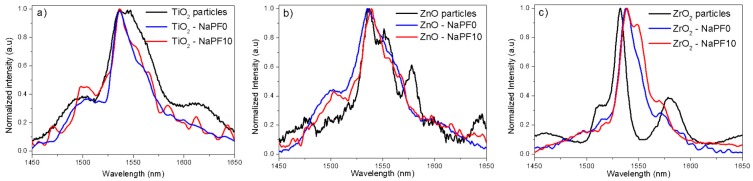
Normalized infrared emission spectra of the Er_2_O_3_-doped TiO_2_ (**a**) ZnO (**b**), and ZrO_2_ (**c**) particles and of their corresponding particles-containing NaPF0 and NaPF10 glasses with 1.25 wt % of particles.

The normalized emission spectra of the NaPF0 and NaPF10 glasses are reported in Figure 6. For the sake of comparison, the spectra of the particles alone are also shown.

Similarly to what previously reported for the NaSrP glasses, the spectra of the NaPF glasses were also different compared to the spectra of the particles alone. However, one can also notice that the shape of the emission depends on the glass composition: the emission band of the NaFP0 glasses was similar to the emission bands presented in Figure 5, while the NaFP10 glasses exhibit different emission bands depending on the particles. It should be pointed out the narrow bandwidth of the emission band of the Er_2_O_3_-doped ZrO_2_ particles-containing NaPF glasses.

**Figure 7:**
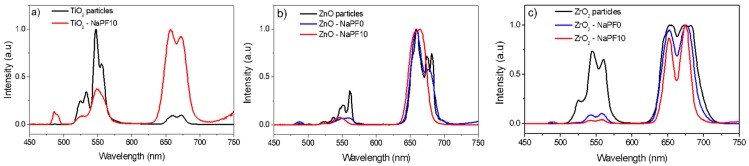
Normalized up-conversion emission spectra of the Er_2_O_3_-doped TiO_2_ (**a**), ZnO (**b**), and ZrO_2_ (**c**) particles and of the corresponding particles-containing NaPF0 and NaPF10 glasses with 1.25 wt % of particles.

In contrast with the SrNaP glasses, the NaPF glasses, except for the TiO_2_-containing NaPF0 glass, exhibit up-conversion emission confirming the survival of the particles. However, as observed for the emission at around 1.5 µm, the shape of the visible emission is modified after adding the particles into the glass (see Figure 7), thus confirming that the site of the Er^3+^ ions in the particles is changed after embedding the particles into the glass. According to Reference [26], the change in the Er^3+^ site can be related to the partial decomposition of the particles during the glass preparation.

The Er^3+^:^4^I_13/2_ lifetime values of the NaPF0 and NaPF10 glasses are listed in Table 1.

Compared to the NaSrP glasses, the NaPF glasses exhibit shorter Er^3+^:^4^I_13/2_ lifetimes. These lifetimes are, however, longer than those of the particles alone. They are also longer than the lifetime of an Er^3+^-doped NaPF10 glass prepared using a standard melting process with Er_2_O_3_ doping level similar to that considered for the particles-containing glasses (Er^3+^:^4^I_13/2_ lifetime equal to (0.6 ± 0.2) ms). Therefore, the local environment of the Er^3+^ ions is expected to be modified after embedding the particles into the glasses. It does not correspond to the local environment of Er^3+^ ions in an amorphous matrix, confirming the survival of the Er^3+^-doped particles into the glasses. Additionally, the Er^3+^:^4^I_13/2_ lifetime depends on the glass composition: independently of the particles, the Er^3+^:^4^I_13/2_ lifetimes of the NaPF10 glasses are the shortest, thus suggesting that the particles are less degraded in the NaPF10 glass than in the NaPF0 one. Similar results were reported in Reference [25].

**Figure 8:**
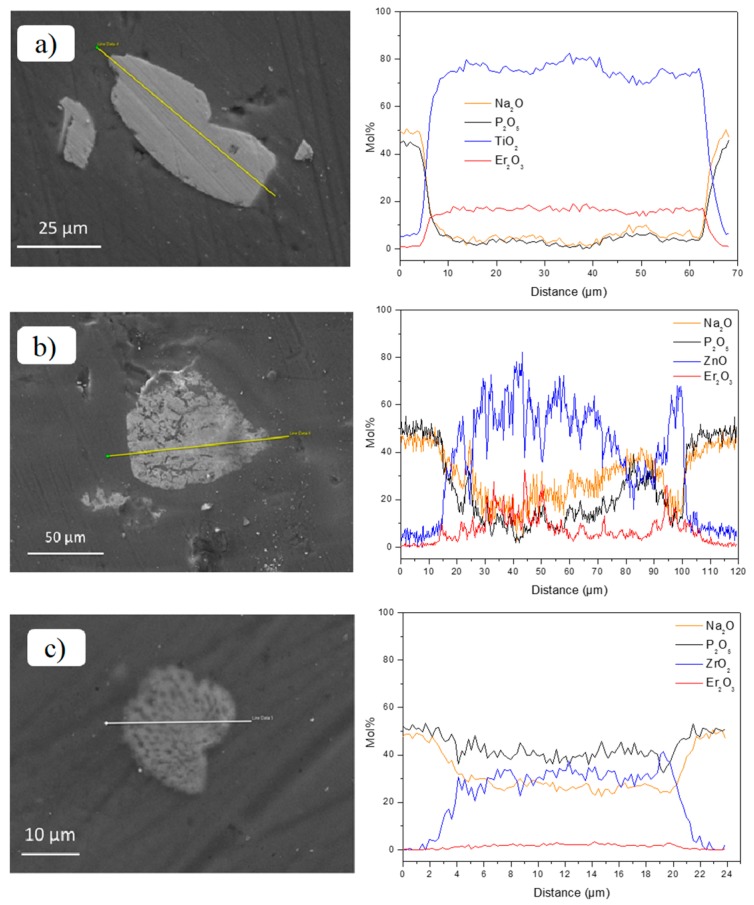
FE-SEM pictures with their corresponding elemental mapping of Er_2_O_3_-doped TiO_2_ (**a**), ZnO (**b**), and ZrO_2_ (**c**) particles-containing NaPF10 glasses. The direction of the scan starts at the circle of the white line (corresponding to 0 µm).

As performed for the NaSrP glasses, FE-SEM/EDS analysis was used to check the morphology and the composition of the particles. In all the glasses, the composition of the glass matrices was found to be in agreement with the theoretical one within the accuracy of the measurement (±1.5 mol %). No crystals around the particles were observed in the glasses, confirming that the crystals formation depends on the glass matrix host (see Figure 8).

Very few TiO_2_ particles were found in the NaPF0 glass, while a large amount of them could be observed in the NaPF10 glass. These particles, as well as the ZnO and ZrO_2_ ones, maintained their compositional integrity in their center in both glasses (see Figure 8), thus confirming the survival of the Er_2_O_3_-doped particles in the glasses as suspected from their spectroscopic properties. However, ~5 mol % of TiO_2_ and ZnO were detected in the glass matrix ~5 µm close to the particles, whereas a lower amount of ZrO_2_ (<3 mol %) was detected around the particles (~1 µm) in the glass matrix. Therefore, as suspected from the color, the shape of the IR and Visible emissions and the Er^3+^:^4^I_13/2_ lifetimes of the particles-containing glasses, the TiO_2_ and ZrO_2_ particles are suspected to degrade the most and the least in the NaPF glasses, respectively, as confirmed by FE-SEM/EDS analysis.

## 4. Conclusions

Particles-containing glasses were fabricated using the direct doping method, which consisted of the incorporation of Er_2_O_3_-doped TiO_2_, ZnO, and ZrO_2_ particles into the glasses after their melting. Although the particles were found to be thermally stable up to 1050 °C, when added at 1000 °C into the glass with composition 50P_2_O_5_-40SrO-10Na_2_O (in mol %), very few ones are suspected to survive, as evidenced by the color (pink or purple depending on the composition of the particles), the FE-SEM/EDS analysis, the broad emission band centered at around 1.5 µm, the absence of the up-conversion and the long Er^3+^:^4^I_13/2_ lifetime of the particles-containing glasses. By lowering the melting temperature and so the doping temperature to 550 °C, it was possible to limit the decomposition of the particles into the glass melt as evidenced by the color of the glasses (light pink or even colorless depending on the particles composition). Particles-containing glasses within the 90NaPO_3_-(10 − x)Na_2_O-xNaF system exhibiting up-conversion were successfully synthesized. From the narrow emission band centered at around 1.5 µm and the Er^3+^:^4^I_13/2_ lifetime values of the particles-containing glasses, the particles, especially the ZrO_2_ ones, are expected to survive in the NaPF10 glass, thus confirming that the direct doping technique can be profitably employed to fabricate novel varieties of glasses with controlled local environment of the Er^3+^ ions when located in crystals.

## Figures and Tables

**Table 1:** Er^3+^:^4^I_13/2_ lifetime values of the Er_2_O_3_-doped TiO_2_, ZnO and ZrO_2_ particles and of the particles-containing NaPF0 and NaPF10 glasses.

Sample Code	Er^3+^:^4^I_13/2_ Lifetime ±0.2 ms		
	Particles Alone	Particles-Containing Glasses	
		NaPF0	NaPF10
Er_2_O_3_-doped TiO_2_ particles	0.1	2.3	0.7
Er_2_O_3_-doped ZnO particles	n.a.	1.4	1.3
Er_2_O_3_-doped ZrO_2_ particles	1.0	1.6	1.4
